# Interdisciplinary collaborative eye examinations to protect preterm infant neurodevelopment: a quality improvement project

**DOI:** 10.3389/fpsyg.2024.1354033

**Published:** 2024-05-06

**Authors:** Dana B. McCarty, Erika Clary-Williams, Kristen D. LeBLond, Tianyi Liu, Tika Zbornik-Thompson, J. Niklas Ulrich, Michelle S. Go

**Affiliations:** ^1^Division of Physical Therapy, Department of Health Sciences, University of North Carolina at Chapel Hill, Chapel Hill, NC, United States; ^2^Pediatric Rehabilitation Services, Monroe Carell Jr. Children’s Hospital at Vanderbilt, Nashville, TN, United States; ^3^Department of Physical Therapy and Occupational Therapy, Duke University Hospital, Durham, NC, United States; ^4^Department of Biostatistics, Gillings School of Global Public Health, University of North Carolina at Chapel Hill, Chapel Hill, NC, United States; ^5^Department of Ophthalmology, University of North Carolina, Chapel Hill, NC, United States; ^6^Department of Ophthalmology, Duke University Medical Center, Durham, NC, United States

**Keywords:** neonatal, eye examination, retinopathy of prematurity, preterm infant, opthalomology, physical therapy, occupational therapy

## Abstract

**Introduction:**

Infants born <31 weeks gestational age with birth weight ≤ 1,500 grams receive routine eye examinations to screen for Retinopathy of Prematurity (ROP) while in the Neonatal Intensive Care Unit (NICU) to help prevent vision threatening complications; however, preterm infants’ sensory systems are underdeveloped, and repeated exposure to painful stimuli is associated with worse developmental outcomes.

**Methods:**

An interdisciplinary NICU team designed a collaborative eye exam model (CEEM) incorporating best practice recommendations for infant pain control during exams. Pain scores and vital signs were recorded before, during, and after exams. Two sets of mixed-effects regression models with a random intercept on infants were established to investigate relationships between the intervention, birth gestational age (BGA), postmenstrual age (PMA), and outcomes associated with painful stimuli. Survey feedback was elicited from NICU stakeholders about the CEEM.

**Results:**

Thirty standard of care (SC) and 35 CEEM exams of 37 infants were included in final analysis. In infants of the same BGA, the number of desaturation events was significantly reduced in the CEEM group (*p* = 0.003) and became 1.53 times smaller with each additional week of BGA (*p* = 0.009). Probability of heart rate recovery within 15 min lowered significantly in the CEEM group (*p* = 0.04). In SC or CEEM or between infants of the same PMA, no differences were observed for bradycardia, heart rate range, chance of heart rate recovery, or pain scores. Increases in tachycardia (*p* < 0.001) events and desaturations *p* = 0.006 were discovered in the CEEM group. When considering interaction effects, the CEEM appeared to reduce the number of desaturations to a greater degree for infants at earliest BGAs with attenuation of this effect with greater BGA. Regarding PMA, bradycardia and tachycardia events were reduced for infants across PMAs in the CEEM, but the effect for tachycardia improves with age, while the effect for bradycardia diminishes with age. Stakeholders agreed that the infant’s eye exam experience and the staff experience was “very much” improved by the CEEM.

**Discussion:**

Despite variable findings in selected outcome measures, the CEEM was positively viewed by staff. Infants may benefit from the CEEM differently based on BGA and PMA.

## 1 Introduction

Retinopathy of prematurity (ROP) is a disease that results from immature vascularization of the preterm infant’s retina and is a leading cause of childhood blindness (Fierson et al., 2018). The American Academy of Pediatrics recommends that preterm infants of ≤1,500 grams or a gestational age of 30 weeks or less receive routine eye exams during hospitalization in the Neonatal Intensive Care Unit (NICU) to screen for this disease so that appropriate treatments and future screening recommendations can be made (Fierson et al., 2018). Due to prolonged hospitalizations in the NICU, especially in the case of extreme prematurity, infants may undergo multiple ROP examinations over the course of hospital stay. While this procedure is necessary to optimize visual function, this procedure can be stressful and painful for preterm infants with limited capacity to self-soothe and modulate pain (Francis, 2016; Disher et al., 2018).

Preterm infants’ sensory systems and self-regulatory pathways are underdeveloped as compared to their full-term counterparts (Knudsen et al., 2021). Infants born less than 34 weeks postmenstrual age (PMA) lack the ability to modulate pain, but pain transmission pathways begin developing during the first trimester (Thill, 2022), allowing infants to experience the “procedural memory of pain” with repeated exposures (Walker, 2019). From preterm birth to term equivalent age, infants experience rapid cortical growth and development, and repeated exposure to painful stimuli during this period has been associated with altered cortical development, worse neurodevelopmental outcomes, and pain sensitivity (Boggini et al., 2021; García-Valdivieso et al., 2023), thermal sensitivity (Hermann et al., 2006), chronic pain (Jones and Psychosocial, 2016) and anxiety in adolescence (Rabbitts et al., 2017). These outcomes vary based on the specific population examined, age at the time of the assessment, and the noxious stimuli presented (Walker, 2019). Therefore, despite well-established negative outcomes associated with early experiences of pain, a lack of standardized protocols and available evidence for infant support during eye exams may contribute to limited implementation of pain reduction strategies across NICUs (Samra and McGrath, 2009; Fajolu et al., 2023).

Evidence suggests that the optimal pain reduction strategy involves multiple modes of developmental support and include: (1) topical anesthetic, (2) oral sucrose, and (3) adjunct interventions such as facilitation non-nutritive sucking, swaddling, and containment (Disher et al., 2018). Adjunct interventions support infant sensory-motor development and can be employed by rehabilitation professionals including physical therapists (PTs) and occupational therapists (OTs) (McCarty et al., 2019; Brinkley et al., 2023). PTs and OTs routinely use pain management strategies (e.g., facilitated tucking) to support infants during routine care and other noxious procedures performed during infant hospitalization (Francisco et al., 2021).

An interdisciplinary collaborative team of therapists, nurses, ophthalmologists, and neonatologists developed a quality improvement (QI) project to improve the consistently of delivering optimal pain reduction strategies in the NICU. The primary objective of this study was to collect infant physiological outcomes both before and after implementation of a collaborative eye exam model (CEEM) using therapist-delivered adjunct interventions for pain control during eye exams. Secondary objectives were to obtain feedback from NICU stakeholders about perceived benefits and challenges of the program on procedural efficiency and infant response to the model. We hypothesized that infants receiving the CEEM would demonstrate reduce adverse events (e.g., oxygen desaturations, bradycardia), reduce infant pain response, and reduce recovery time for return to baseline vitals across chronological and postmenstrual age. We also hypothesized that infants born earlier (i.e., of lower birth gestational age) may demonstrate less benefit from the CEEM due to potentially greater respiratory comorbidities, and that infants of the same postmenstrual age, regardless of their birth gestational age (BGA), would respond similarly to the intervention based on potentially similar levels of neurobehavioral maturity.

## 2 Materials and methods

### 2.1 Participants and setting

This QI project was conducted in the Neonatal Critical Care Center at UNC Children’s Hospital, a Level IV NICU where approximately 150 infants <31 weeks BGA are cared for annually. The project was approved by the UNC Institutional Review Board. A total of 48 unique patients underwent a total of 102 eye exams between January–April 2021. Racial and ethnic demographic information was not collected for the purposes of the QI project; however, typical racial and ethnic distributions for the UNC Newborn Critical Care Center preterm infant population are as follows: Black or African American (30%), white (65%), more than one race (5%), Hispanic (6%), with distribution of sex as 50% male and 50% female. Inclusion criteria were as follows: preterm infants who met ROP screening criteria (Fierson et al., 2018) with active PT and OT orders (i.e., BGA <32 weeks and < 1,500 grams at birth). Exclusion criteria were as follows: infants who were determined by the medical staff to be inappropriate for eye exams based on infant medical status (e.g., tenuous respiratory status, high frequency jet ventilation requirements).

Infants undergoing eye exams were > 32 weeks PMA (96.1%), and < 4% of infants were 31 weeks PMA at the time of the exam. A total of 102 independent ROP exams were completed with 43 exams in the pre-intervention group and 59 in the post-intervention group; however, a retina fellow participated in 9 ROP exams in the pre-intervention group and 19 ROP exams in the post-intervention group. Due to the increased time and potentially increased infant stress with 2 examiners, these 28 exams were excluded from analysis.

Measurements were collected over a total period of 12 weeks. Two attending ophthalmologists (JNU, MSG) conducted 6 weeks of ROP exams each, which included 3 weeks of the standard of care phase and 3 weeks of the CEEM phase. The study was conducted in this manner to reduce bias from ophthalmologist approach, with each ophthalmologist conducting 3 weeks of eye exams in standard of care and 3 weeks in the CEEM phase. The infant’s bedside nurse, or any available nurse, provided developmental support during the standard of care (SC) phase. The same 2 neonatal therapists, the principal investigator (DM) with advanced level training in neonatal PT, and one enrolled in a post-graduate fellowship in neonatal physical therapy who was trained by the PI (EC-W), provided developmental support during the CEEM phase.

### 2.2 Eye examination procedure

One of two qualified ophthalmologists (JNU, MSG) performed dilated fundus examinations with binocular indirect ophthalmoscopy using an eyelid speculum and a 28-diopter lens (Volk Optical, Mentor, OH) and scleral depression using either a CalgiSwab (Puritan Medical Company, Guilford, ME) or Schocket double ended scleral depressor (Bausch and Lomb, Vaughan, Ontario, Canada).

#### 2.2.1 Standard of care for developmental support during eye exam

##### 2.2.1.1 Prior to exam

In standard of care ROP exams in the unit, all infants scheduled for ROP screening receive dilating drops (cyclopentolate 0.2%-phenylephrine 1% combination 1 drop given every 5 min for 3 doses, or cyclopentolate 0.5% 1 drop and phenylephrine 2.5% 1 drop given every 5 min for 2 doses for infants requiring stronger mydriasis) 1–2 h prior to ROP examination. All infants receive proparacaine 0.5% topical anesthetic drops at least approximately 30 s prior to placement of an eyelid speculum for their examination. The ophthalmologist usually conducted exams over a period of 1–2 h depending on the number of infants being examined beginning between 7:30 am and 10:00 am depending on ophthalmologist availability and schedule.

##### 2.2.1.2 Set up

When the ophthalmologist entered the infant’s room, he or she requests nursing assistance to provide developmental support and attendance at the bedside during the exam. Any available room nurse who was not participating in medical team rounds, performing routine infant care, or feeding an infant, would provide assistance as able. The infant was repositioned so that the head is easily accessed by the examiner, often requiring a 90-degree whole body turn of the infant, rotation into supine position (if not currently supine), and flattening of the head of the bed.

##### 2.2.1.3 Infant support

Infants were tightly swaddled with hands away from the infant’s face, often down at the infant’s side, to prevent reaching and disruption of the exam. The nurse held the infant’s body with one hand to promote stillness and containment and cupped the head in the other hand in an effort to both support the infant and expedite the exam process. The nurse may have used sucralose if appropriate by delivering drops to the corner of the infant’s mouth during the exam, and may have used a pacifier if available and supportive (Disher et al., 2018). Medical supports that the nurse may have used during the exam include stopping nasogastric tube feedings, increasing oxygen support, and providing pain medicine immediately before or after (Disher et al., 2018; Tan et al., 2019). Nurses may have employed environmental supports such as turning down overhead lights, using auditory stimulation (soft whispers, bedside music), and developmental pacing (taking breaks as the infant displays stress signs). The nurse monitored the infant’s vital signs and may suggest taking breaks from the eye exam based on his or her medical judgment. Nurses providing developmental support had varying levels of training in developmental care and inconsistent delivery of such supports. There were no standards in the NICU for how developmental support should be provided during ROP exams.

##### 2.2.1.4 Recovery

When the eye exam concludes, the nurse repositioned the infant to their original position, makes any necessary adjustments to the infant’s oxygen and/or feeding delivery, and provides calming strategies to the infant as time allows.

#### 2.2.2 Collaborative eye exam model

The Collaborative Eye Exam Model (CEEM) was developed to address 2 main concerns raised by the NICU’s developmental care committee related to ROP examinations. The first concern was that infants were not receiving optimal pain support based on inconsistent delivery of non-pharmacologic and developmentally-appropriate pain control strategies (Disher et al., 2018). These variations were the result of (1) nurse availability at the time of the exam and (2) the individual nurse’s level of training in best practices non-pharmacological and developmentally-appropriate pain control strategies. The second concern was the lack of a standardized process for conducting eye exams in the unit, and as a result, expectations about roles varied among staff. Therefore, the CEEM established clear roles and expectations among staff by bringing in designated therapists to provide infant support in a consistent manner, which allowed nursing staff to attend to other infant needs in the unit while still being available to intervene for the infant’s medical needs if necessary.

##### 2.2.2.1 Prior to exam

As in SC ROP exams, all infants scheduled for ROP screening received dilating drops 1–2 h prior to examination. Prior to the ophthalmologist’s arrival, two therapists visit each infant’s bedside nurse to determine best timing for exam based on timing of eye drop administration and other procedures that the infant might have that morning. The therapist also inquired about any additional medical supports (need to stop feedings, increase oxygen support, etc.) that the nurse recommended and used this information to organize the order of eye exams as it meets the needs of the infants on the unit while optimizing efficiency. Topical anesthetic was administered as described in 2.2.1.

##### 2.2.2.2 Set up

Two therapists rounded with the ophthalmology team and alternate providing developmental support to the infant. The therapist located the bedside nurse, informed them that the eye exam was about to begin and requests any medical support(s) necessary. The therapist then repositions the infant in preparation for the exam as described above.

##### 2.2.2.3 Infant support

Infants were tightly swaddled, but, in contrast to extended upper extremity positioning down at sides, the upper extremities were allowed to flex and cross the upper chest. This body position is most likely to limit the infant’s associated movements based on “motor reactivity” because the fetal position is being emulated (Francisco et al., 2021) with support of one of the therapist’s hands, preventing startle. With the other hand, the therapist cupped the infant’s head in midline and adjusted head position based on ophthalmologist’s direction of approach to expedite the exam. Therapists regularly used oral sucralose if the infants accepted a pacifier and employed environmental supports including low lighting and soft auditory support. The therapist monitored the infant’s vital signs and suggested breaks if the infant’s experienced bradycardia, had a heart rate of >200 bpm, or demonstrated desaturation that did not recover spontaneously. The nurse was available in the room and at therapist request to implement any medical supports as described above (e.g., stopping nasogastric tube feedings, increasing oxygen support, and providing pain medicine immediately before or after).

##### 2.2.2.4 Recovery

When the eye exam concludes, the therapist providers developmental support until the infant’s vital signs return to baseline. This may include re-swaddling or repositioning the infant within their blanket or positioning device, holding/picking up the infant, and/or providing vestibular input (e.g., rocking, bouncing). The therapist updates the nurse at the end of the exam.

The CEEM intervention is outlined in greater detail using the Template for Intervention Description and Replication, (TIDieR) Criteria in Appendix A in Supplementary material (Hoffmann et al., 2014).

### 2.3 Outcome measures

The Premature Infant Pain Profile (PIPP) was used to determine the infant’s pain response during the exam. The PIPP is commonly used in this population to assess procedure-related pain. The score ranges from 0 to 21 and accounts for facial behaviors, heart rate and oxygen level, and fixed variables (gestational age and baseline behavioral state) to quantify mild (0–6), moderate (7–12), or severe pain (>12). A NICU intern trained by a neonatal therapist (EC-W) collected pain scores during the exam.

The following vital signs were collected via ECG leads and pulse oximeter as displayed on the infant’s monitor at the time of the eye exam by the NICU intern: peak heart rate, lowest oxygen saturation, and number of bradycardia and desaturation events. Time for the ophthalmologist to complete the exam was recorded in minutes by the NICU intern based on the monitor display. Total minutes were recorded instead of seconds to align start time with the monitor time used for recording vitals. Following the eye exam, the number of bradycardia, tachycardia, and desaturation events were recorded at 15-min intervals up to 2 h after the exam. Heart rate range was calculated as the difference between the lowest and highest heart rates recorded during the infant’s eye exam. We determined if heart rate recovery had taken place if the infant’s average HR at the first 15 min interval was smaller than or equal to the infant’s heart rate at the start of the exam. A neonatal therapist (EC-W) collected data from the monitor post-exam by accessing data on each infant’s monitor history.

Infant demographic information including BGA and PMA at time of exam were collected. No other demographic variables were collected for analysis.

A digital survey consisting of 8 questions was disseminated via email to all nurses, physicians, nurse practitioners, therapists, and ophthalmologists in the unit. Additionally, one member of the parent advisory board for the unit who had observed standard of care eye exams was invited to participate in the survey. The questions gathered data about the individual’s discipline, familiarity with standard of care eye exams and the CEEM, as well as advantages and challenges with both processes. See Appendix B in Supplementary material.

### 2.4 Statistical analysis

**Figure 1:**
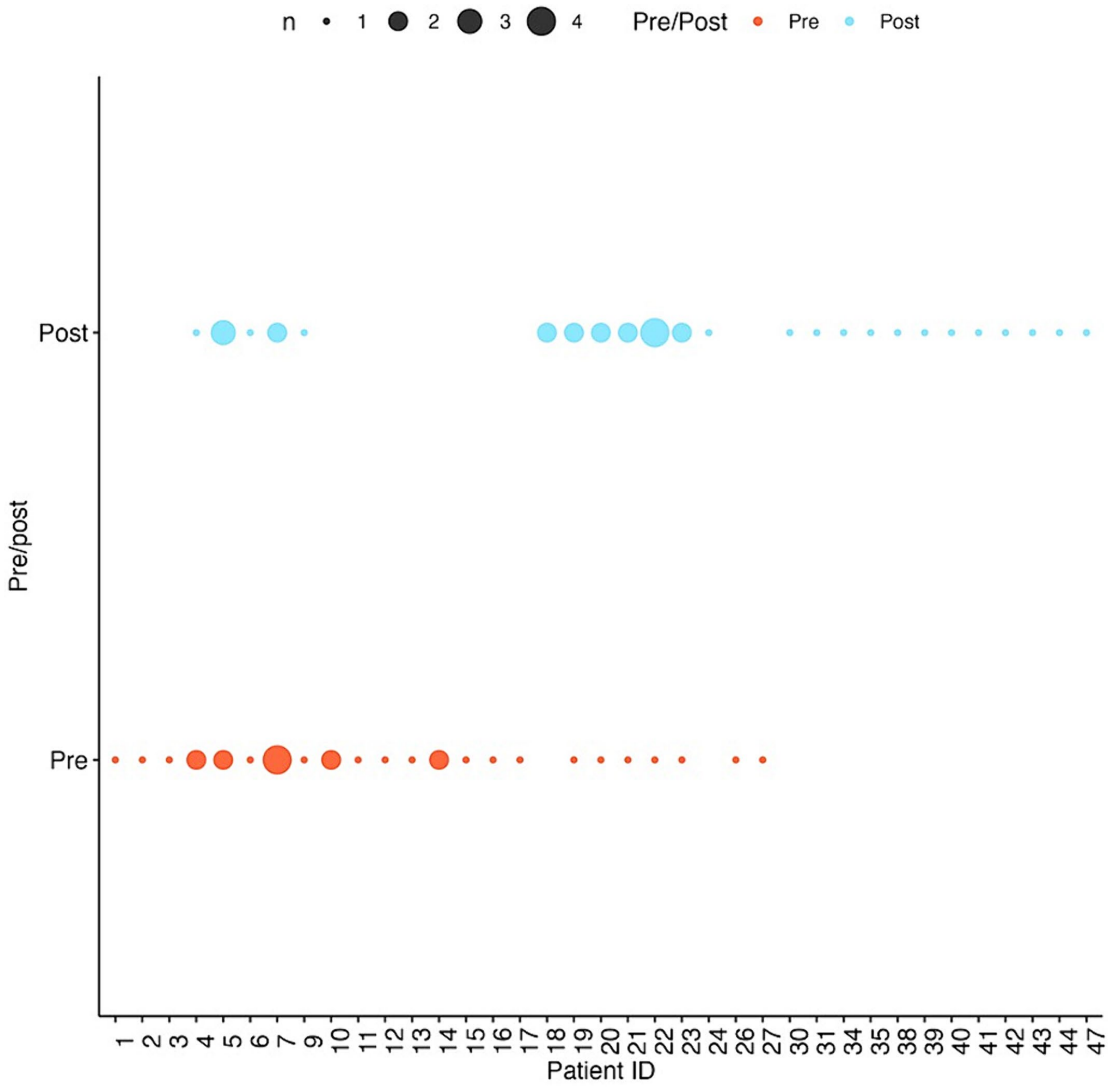
Number of visits per infant patient pre-and post-intervention. Larger dots correspond to more visits by the same patient.

A total of 35 SC exams and 40 CEEM exams were performed by a single ophthalmologist examiner. Within the study period, individual infants underwent a range of 1–4 eye exams (Figure 1). To reduce confounding effects, 4 infants with the highest comorbidity score (sum of the risk factors bronchopulmonary dysplasia, intraventricular hemorrhage, stage 4 or 5 retinopathy of prematurity, and any neonatal infection, shown to be a predictor of poor neonatal outcomes) were excluded, resulting in 30 SC exams and 35 CEEM exams of 37 infants included in final analysis.

On the data of included exams, two sets of mixed-effects regression models with a random intercept on infants were established to characterize relationships between the administering of the ROP exam, BGA, PMA, and adverse events of the infants’ vital signs associated with painful stimuli: one set with the intervention indicator, BGA, and their interaction as covariates, and the other with the intervention indicator, PMA, and their interaction. Mixed-effects Poisson regression models, a natural choice for modeling the frequencies of adverse events (Sun et al., 2020), were employed for bradycardia, tachycardia, and desaturation counts during exams with the aforementioned covariates. Linear mixed-effects model and mixed-effects logistic regression models were used for characterizing the pain scores and the occurrence of infant heart rate recovery after 15 min, respectively. Details of the model parameters and their roles in characterizing the outcomes are given in the next section. A discussion of the interpretation of the model parameters for non-random covariates have been included in the Discussion section.

### 2.5 Model parameters

We now briefly introduce the mathematical specification of the mixed-effects models. The calculation of the results in Section 3.2 and 3.3 hinge directly on this specification. For Mixed-effects Poisson regression models, we model the logarithm of the expected adverse event (AE) counts through the followings:


$$\begin{array}{l} BGA:\log\left(\mathrm{Expected}\;AE\;\mathrm{coun}t\right)=\beta_{0}+b_{i}+\beta_{1}*BGA\\ \;+\beta_{2}*I\left(\mathrm{CEEM}\right)+\beta_{3}*BGA*I\left(\mathrm{CEEM}\right)\text{.} \end{array}$$

$$\begin{array}{l} PMA:\log\left(\mathrm{Expected}\;AE\;\mathrm{count}\right)=\beta_{0}+b_{i}+\beta_{1}*PMA\\ \;+\beta_{2}*I\left(\mathrm{CEEM}\right)+\beta_{3}*PMA*I\left(\mathrm{CEEM}\right)\text{.} \end{array}$$

Here, $I\left(\mathrm{CEEM}\right)$ is an indicator variable, taking the value of 1 if the CEEM is applied and 0 if the SC exam is applied, while $b_{i}$ is the subject-specific random intercept. In the generalized mixed-effects model context, the logarithm function on the left hand side of the equation is called the “link function.” To characterize the expected adverse event counts, we use the estimates of the non-random parameters (Table 1). We add a hat to each parameter to denote its estimate (i.e., ${\hat{\beta }}_{2}$ is the estimate for $\beta_{2}$ ). In the other two types of models, the specification of the parameters are the same as in the Poisson model above, with the only difference being the link function. The linear regression model has the “identity” link function, meaning the left hand side is just the expected pain scores, while the logistic regression model adopts a “logit” link function $\log\left(p/(1-p\right))\text{,}$ where *p* is the probability of heart rate recovery in 15 min.

**Table 1:** Intervention and interaction effects by birth gestational age and postmenstrual age.

Birth gestational age (BGA)						Postmenstrual age (PMA)					
	Estimate	Standard error	*Z*-value	Pr (>*z*)			Estimate	Standard Error	*z*-value	Pr (>*z*)	
Bradycardia						Bradycardia					
(Intercept)	6.811	5.053	1.348	0.178		(Intercept)	−1.23	4.27	−0.29	0.77	
BGA	−0.297	0.191	−1.554	0.120		PMA	−0.00	0.12	−0.02	0.99	
Group effect	−5.120	4.355	−1.176	0.240		Group effect	−7.002	4.85	−1.45	0.15	
BGA × Pre-post interaction effect	0.165	0.172	0.956	0.339		PMA × Pre-post interaction effect	0.17	0.13	1.28	0.20	
Tachycardia						Tachycardia					
(Intercept)	−6.175	8.982	−0.687	0.49		(Intercept)	−32.45	8.48	−3.82	<0.00	
BGA	0.117	0.311	0.376	0.707		PMA	0.77	0.22	3.54	<0.00	
Pre-post intervention effect	−3.507	4.467	−0.785	0.433		Pre-post intervention effect	44.97	9.38	4.89	<0.00	
BGA × group interaction effect	0.143	0.163	0.874	0.382		PMA × group interaction effect	−1.26	0.26	−4.75	<0.00	
Desaturations						Desaturations					
(Intercept)	11.75	4.50	2.61	0.009		(Intercept)	−3.50	1.39	−2.52	0.01	
BGA	−0.43	0.17	−2.60	0.009		PMA	0.1	0.04	2.88	0.004	
Group effect	−6.44	2.19	−2.95	0.003		Group effect	3.92	1.42	2.77	0.006	
BGA × group effect	0.27	0.09	3.09	0.002		PMA × group effect	−0.09	0.04	−2.5	0.01	
Heart rate recovery						Heart rate recovery					
(Intercept)	0.761	5.12	0.15	0.88		(Intercept)	−9.65	7.98	−1.21	0.23	
BGA	0.01	0.19	−2.03	0.04		PMA	0.30	0.22	1.34	0.18	
Group effect	−15.29	7.55	−2.03	0.04		Group effect	9.34	9.01	1.04	0.30	
BGA × group interaction effect	0.56	0.28	1.98	0.05		PMA × group interaction effect	−0.28	0.25	−1.10	0.27	
	Estimate	Standard error	df	*t*-value	Pr (>t)		Estimate	Standard error	df	*t*-value	Pr (>t)
Heart rate range						Heart rate range					
(Intercept)	128.22	52.77	47.48	2.43	0.02	(Intercept)	12.26	60.77	59.99	0.20	0.84
BGA	−2.46	1.93	49.40	−1.28	0.21	PMA	1.32	1.66	59.97	0.80	0.43
Group effect	25.05	64.15	58.18	0.39	0.70	Group effect	16.86	70.87	53.58	0.24	0.81
BGA × group interaction effect	−0.98	2.37	59.11	−0.41	0.68	PMA × group interaction effect	−0.48	1.94	52.74	−0.25	0.81
	Estimate	Standard error	df	*t*-value	Pr (>t)		Estimate	Standard error	df	*t*-value	Pr(>t)
Pain scores						Pain scores					
(Intercept)	13.54	5.77	46.72	2.35	0.023	(Intercept)	14.41	6.01	59.12	2.40	0.20
BGA	−0.02	0.21	48.06	−0.11	0.91	PMA	−0.40	0.17	59.24	−0.24	0.81
Group effect	−0.26	6.3	53.89	−0.04	0.97	Group effect	−1.15	6.87	48.69	−0.168	0.87
BGA × group interaction effect	−0.00	0.24	56.21	−0.01	0.99	PMA × group interaction effect	0.02	1.19	47.50	0.11	0.91

#### 2.5.1 Condition effects

In a Poisson mixed-effects model, comparing the incidence rate between two infants of the same BGA or PMA who received different interventions, as in section 3.2, we have


$$\log\left(\mathrm{Ratio}\;\mathrm{of}\;\mathrm{expected}\;AE\;\mathrm{counts}\right)={\hat{\beta }}_{2}\text{.}$$

Hence, a positive ${\hat{\beta }}_{2}$ indicates an increase in the expected AE counts in infants receiving the CEEM versus those given the SC exam, signifying an increase in the likelihood of having AEs, while a negative ${\hat{\beta }}_{2}$ indicates the opposite. The ${\hat{\beta }}_{2}$ in other two types of models have a similar interpretation, with a positive value indicating an increase in pain score (linear mixed-effects regression) or an increase in the likelihood of heart rate recovery and a negative value indicating the opposite.

#### 2.5.2 Interaction effects

Interaction effects delineate the additional effects on the outcomes on top the main effects by the BGA/PMA and the CEEM. To see this, assume we have an infant of 32 weeks of BGA receiving the CEEM, and we wish to compare this infant’s expected number of bradycardias to that of another infant of 31 weeks of BGA but receiving the SC exam. The interaction effects between BGA and the CEEM (on the logarithm of ratio of expected AE counts) is then


$${\hat{\beta }}_{3}*\left(32-31\right)*\left(1-0\right)={\hat{\beta }}_{3}\text{.}$$

Interaction effects are better interpreted in combination with the main effects. The logarithm of the ratio of expected AE counts between same two infants are


$$\begin{array}{l} \log\left(\mathrm{Ratio}\;\mathrm{of}\;\mathrm{expected}\;AE\;\mathrm{counts}\right)={\hat{\beta }}_{1}*\left(32-31\right)\\ +{\hat{\beta }}_{2}*\left(1-0\right)+{\hat{\beta }}_{3}*\left(32-31\right)*\left(1-0\right)={\hat{\beta }}_{1}+{\hat{\beta }}_{2}+{\hat{\beta }}_{3}\text{.} \end{array}$$

Same as the condition effects described the last section, the interaction effects in the other two types of models have a similar interpretation.

#### 2.5.3 Fitted values of outcome variables

**Figure 2:**
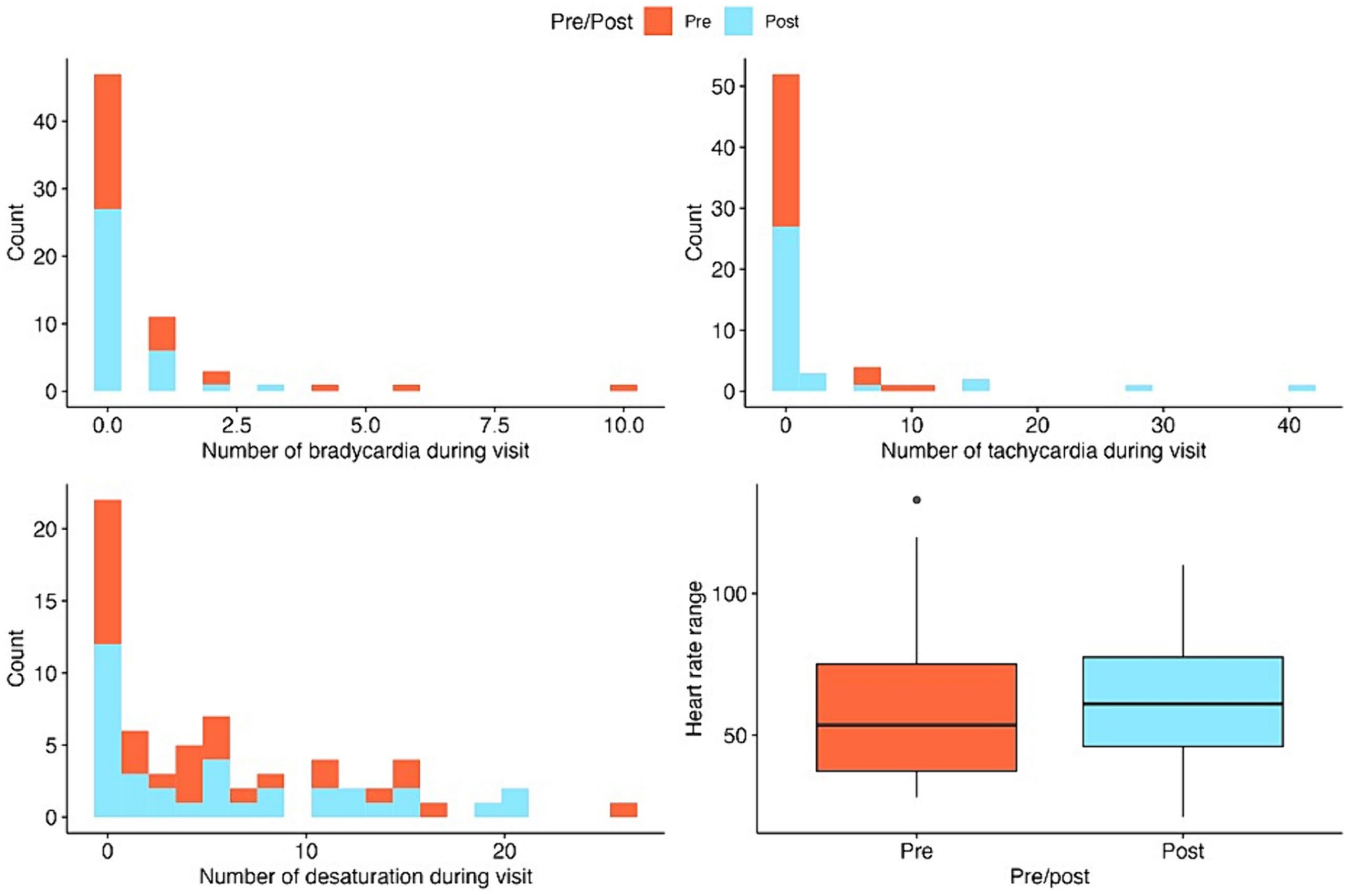
Stacked histograms and boxplots for the summaries of key outcome variables pre-and post-intervention. Histograms are used for count outcomes and boxplots for continuous outcomes.

In this section, we describe how the fitted values in Figure 2 (shown in black dots and black lines) are obtained. In short, the fitted values are the expected number of AEs, or expected pain score, or expected probability of recovering heart rate after 15 min, for an infant of a given BGA/PMA and CEEM/SC group status based on the estimates for the non-random parameters in the model specified above. Mathematically, denote the following value as *x*:


$$x={\hat{\beta }}_{0}+{\hat{\beta }}_{1}*PMA+{\hat{\beta }}_{2}*I\left(\mathrm{CEEM}\right)+{\hat{\beta }}_{3}*PMA*I\left(\mathrm{CEEM}\right)\text{.}$$

Then the fitted values for each type of mixed-effects model are obtained through.

Poisson regression: $e^{x}$ ; this yields the estimated expected AE count of the infant.

Linear regression: x ; this yields the estimated pain score of the infant.

Logistic regression: $\frac{e^{x}}{1+e^{x}}$ ; this yields the estimated probability of heart rate recovery in 15 min of the infant.

## 3 Results

### 3.1 Group differences

Of the infants included in the analysis, the average BGA was 28.06 weeks (standard deviation, 2.29, range 22.4–33.2), and average PMA at the first ROP exam was 34.81 weeks (standard deviation 2.37, range 31.2–40.6). See Table 2 for summary statistics of key demographic and vital sign outcome variables for the SC and CEEM groups.

**Table 2:** Summary statistics for key demographic and vital sign outcome variables in the analysis.

	Pre-intervention (*n* = 30)	Post-intervention (*n* = 35)
BGA – first visit	27.72 (2.40)	27.58 (2.44)
PMA – first visit	35.76 (2.27)	34.66 (2.44)
BGA – overall	27.10 (2.45)	26.82 (2.64)
PMA – overall	36.60 (2.93)	35.70 (3.43)
Pain Profile of Premature Infant Scores (PIPP)	13.10 (2.37)	12.40 (2.67)
Total bradycardia	0.97 (2.17)	0.31 (0.67)
Total tachycardia	1.27 (2.79)	3.23 (8.73)
Total desaturation	5.43 (6.55)	6.03 (6.57)

The mean for each variable is reported separately for 30 pre-and 35 post-intervention visits. The associated standard deviation is reported between the parentheses. BGA, birth gestational age; PMA, postmenstrual age. The means and standard deviations of BGA and PMA are reported separately for infants at their first visit and overall due to multiple exams on some infants.

We ran two sample *t*-tests to examine potential differences between groups and determined that the groups were similar based on *p*-values >0.05 for PMA at the time of the exam (*p* = 0.7) and resting heart rate taken prior to the exam (*p* = 0.99).

### 3.2 Group effects

For SC and CEEM groups, the numbers of bradycardia events, tachycardia events, heart rate range, pain scores did not differ.

#### 3.2.1 Birth gestational age

In infants of the same BGA, the number of desaturation events was significantly reduced in the CEEM group (*p* = 0.003) and became 1.53 times smaller with each additional week of BGA (*p* = 0.009). Probability of heart rate recovery within 15 min lowered significantly in the CEEM group (*p* = 0.04).

#### 3.2.2 Postmenstrual age

For SC and CEEM groups or between infants of the same PMA, no differences were observed for bradycardia, heart rate range, chance of heart rate recovery, or pain scores. Increases in tachycardia (*p* < 0.001) events and desaturations *p* = 0.006 were discovered in the CEEM group (Table 1).

### 3.3 Interaction effects

#### 3.3.1 Birth gestational age × group

The interaction effect of the group and BGA did not demonstrate significant differences in the number of bradycardia events, tachycardia events, heart rate range, or pain scores. The interaction effect of BGA and group on desaturation events demonstrated results in the opposing direction of group effects such that for each one-week increment in BGA in the CEEM group, there was an expected 1.32-time increase in desaturation events (*p* = 0.002). The interaction effect of group and BGA on heart rate recovery was such that the probability of infants recovering their heart rate after 15 min increases in the CEEM group for each incremental week in BGA (*p* < 0.05).

The conditions of the CEEM group appeared to reduce the expected number of desaturations to a greater degree for infants born at the earliest BGAs (i.e., 24 weeks). As the BGA approached term equivalent age (40 weeks) the effects of reduction were weaker. Additionally, the CEEM group condition may improve heart rate recovery in infants with BGA of 28 weeks or more, but was less likely to improve heart rate recovery for infants with BGA <28 weeks.

#### 3.3.2 Postmenstrual age × group

The interaction effect of group and PMA did not demonstrate significant differences in the number of bradycardia events, heart rate range, probability of heart rate recovery, or pain scores; however, interaction effects demonstrated that the expected number of desaturations became 1.10 times smaller for each one-week increment in PMA in the CEEM group (*p* = 0.012) (Table 1).

Bradycardia and tachycardia events were reduced for infants across the PMA range in the study, but the effect for tachycardia improved with age, while the effect for bradycardia diminished with age. Finally, the condition of the CEEM group helped to reduce the desaturation events for infants at or near-term equivalent age (i.e., 40 weeks PMA).

**Figure 3:**
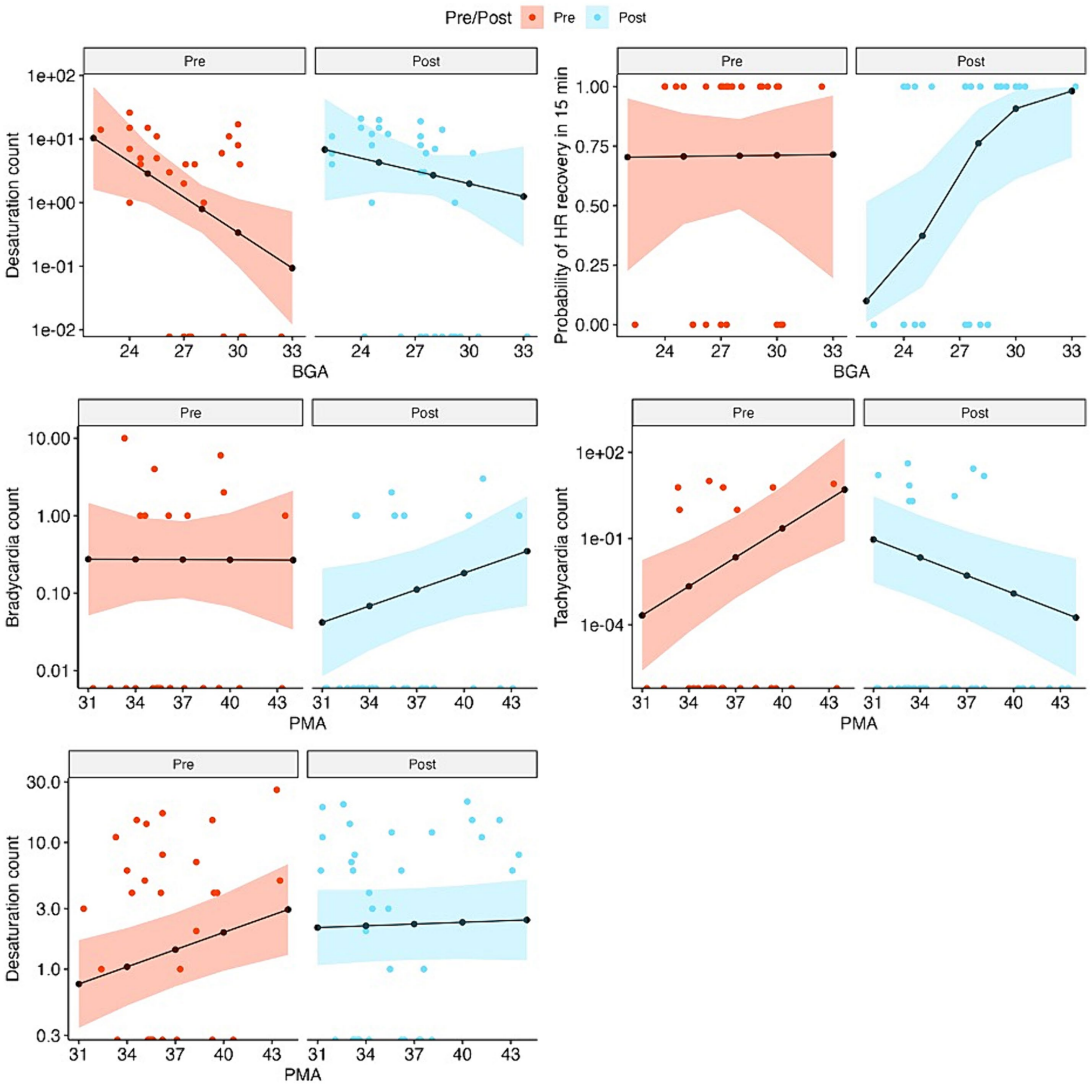
Observed and fitted values of outcome variables from the fixed effects of the mixed-effects models, separately for pre-and post-intervention visits, for outcomes that are associated with significant BGA or PMA effects. Observed values are in colored dots and fitted values are in black lines and dots. The colored bands show 95% confidence intervals of the fitted values. Count data are plotted on the log scales and the values on the *x*-axis represent zeroes.

Plots in Figure 3 demonstrate expected count outcomes for SC and CEEM groups at various BGA and PMAs within 95% confidence interval bands and aid in our interpretation of the above results.

### 3.4 Survey responses

**Figure 4:**
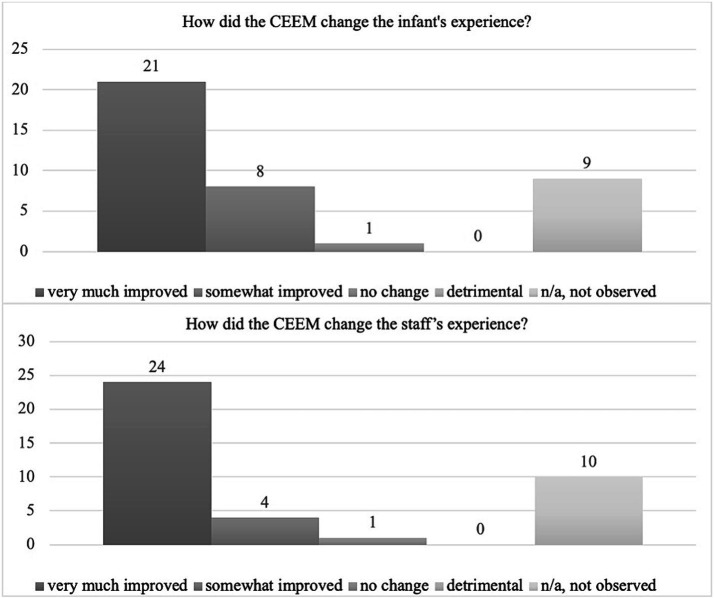
Stakeholder survey results.

A total of 39 stakeholders (23 nurses, 5 therapists, 8 neonatal physicians or nurse practitioners, 1 parent) completed the survey. Seventy-two percent of stakeholders had observed both standard of care and collaborative eye exam processes. The majority reported that they felt that CEEM either very much (54%) or somewhat (21%) improved the infant’s eye exam experience and very much (62%) or somewhat (10%) improved the staff’s experience. No negative comments regarding infant or staffing experience were recorded (Figure 4).

The following example statements of responders reporting a positive infant experience:

“I have noticed babies having less brady(cardias)/desat(urations) with having the therapists contain both during the exam as well as after. The time of the eye exam has also seemed to go significantly faster.” – Neonatal nurse

“Overall, I felt the infants were quicker to calm when therapy participated than when they did not.” – Therapist

The following example statements of responders reporting a positive staff experience:

“Eye exams are always in the morning at the worst times; during cares, during rounds, etc. Having the therapists there to get them adjusted and not have to step away for any reason was incredibly helpful!” – Neonatal nurse

“I loved having neonatal (physical and occupational) therapy because it made rounds run more smoothly. Each baby was ready quicker … The baby received post-exam care, which helped the baby calm down back to baseline.” – Ophthalmologist

Despite overall positive findings from the survey, 9/39 (23%) and 10/39 (26%) of stakeholders reported they had not observed both the standard of care and CEEM for comparison (Figure 4).

## 4 Discussion

Our QI project examining a collaborative approach to neonatal eye exams resulted in variable outcomes, which is consistent with previous studies examining developmentally supportive interventions. Overall, the results demonstrate that infants may benefit from the CEEM based on their BGA, PMA at time of exam, and particular outcome of interest. The variability of infant response based on age at time of the exam is consistent with previous studies examining neonatal pain interventions (Samra and McGrath, 2009; Walker, 2019).

While there is consensus about best practices for pharmacological interventions to reduce pain during neonatal eye exams (Pirelli et al., 2019; Thirunavukarasu et al., 2022), there is no consensus for supportive nonpharmacological interventions that should be employed. This lack of consensus is in part, due to variable interventions, outcomes, and populations examined. We experienced difficulty describing what the unit’s standard of care practices were because of highly variable nursing practices used to reduce infant pain during ROP exams.

We did not find significant differences in pain according to the PIPP between groups in our study, which is consistent with findings of studies examining similar developmental interventions in preterm infants (Rush et al., 2005; Kleberg et al., 2008; Disher et al., 2018). However, some studies have noted small improvements in pain response with developmental interventions. Metreş and Yıldız (2019) found that the “ROP position,” which consists of a 2-person facilitation of head immobilization, arms flexed toward head and lower extremities flexed in midline, reduced pain and shortened crying periods in infants with a range of BGAs from 28 to 36 weeks. Sun et al. (2020) found that “Gentle Human Touch,” which consists of head cupping and arm containment, during eye exams reduced pain in infants <34 weeks BGA, and Boyle et al. (2006) determined that PIPP scores were lower in infants <32 weeks BGA using a pacifier during ROP exams.

There are many ways that pain may be experienced or exacerbated during the neonatal eye exam. Findings support that infant pain scores are highest during initial insertion of the speculum (Gal et al., 2005), and that infant pain increases with longer exam times (Corrigan et al., 2020). Therefore, pain reduction may be accomplished through multiple mechanisms that include pharmacological analgesics prior to and oral sucrose at the time of speculum insertion, both of which were employed in our study. Additionally, skilled therapists as part of a practiced team of clinicians not only employ developmental positions as described above, but also may learn and adjust to ophthalmologist individual approaches to ROP exams. The increased competence gained from using a consistent eye exam team while navigating space constraints and individual infant needs may provide more efficient support, ultimately reducing the time that infants are experiencing pain.

A potential concern with using pain scales in the preterm infant population is the spectrum of developmental responses to pain across PMA. Very ill and preterm infants are less robust in their stress behaviors. Despite both the original PIPP and revised PIPP (PIPP-R) having mechanisms to account for PMA at time of exam (Stevens et al., 2014), pain scales are more likely to capture vigorous responses to pain (Zeiner et al., 2016) that are less prevalent in very ill or very preterm infants. Furthermore, while most studies examining ROP exam-related pain have used the PIPP, only one study by Chuang et al. (2019) which found that a modified developmental care bundle improved pain and stress responses in infants born <30 weeks, has used PIPP-R; therefore, consistent use of objective measures of pain are necessary to accurately compare outcomes across studies. This variability in pain measurement has led some researchers to suggest exploring other physiological measures of pain (e.g., heart rate variability) to more objectively measure pain response in preterm infants (Gibbins et al., 2008). Additionally, accurate measurement of PIPP scores may be challenging based on the observer’s ability to view the infant’s body, face, and monitors during the exam. For this reason, Boyle et al. (2006) only recorded pain response during the exam of the first eye only because the ophthalmologist blocked the observer’s view for the second eye. Considerations such as these, along with the moment that PIPP scores were recorded (e.g., before, during, after), are quite variable among studies.

Outcomes related to adverse events during ROP exams are also somewhat variable (Disher et al., 2018). While use of oral sucrose is supported as a nonpharmacological pain reduction intervention (Gal et al., 2005). Grabska et al. (2005) found that infants experienced a small but significant drop in oxygen saturation with administration of oral sucrose. Therefore, infants may response differently to oral sucrose delivery, and use of this kind of support during eye exams may be infant-dependent. In our own study, sucrose was only provided to infants who readily accepted a pacifier, an indication that the suck-swallow-breathe reflex is developing or intact (Lubbe and Ten Ham-Baloyi, 2017); however, there is a need to more clearly define which infants benefit from oral sucrose interventions based on current level of respiratory support, whether a pacifier is used to supplement sucrose delivery, and PMA at time of exam.

Use of “nesting,” or using swaddling blankets and boundaries to provide circumferential support, is hypothesized to improve pain by limiting infant motor responses (e.g., arm and leg movement), and similarly, use of a pacifier is also hypothesized to reduce pain by limiting crying, but overall support is weak (Kandasamy et al., 2011). For example, Slevin et al. (1997) noted significantly less distress as measured by infant crying and activity in infants who were nested versus those who were not, but no physiological measures were found to be statistically different between groups.

While nurses in our study as well as previously published work reported negative physiological responses in infants both during and following eye exams (Tan et al., 2019), a recently published study by Sullivan et al. (2022) that examined 5 years of infant data 24 h before and after ROP exams found that most very low birthweight infants had no increase in significant cardiorespiratory events in response to ROP exams; however Onuagu et al. (2022) found a 2-fold increase in sympathetic activation as measured by skin conductance during and after ROP exams in preterm infants, and propose that adverse events during and after ROP exams are widely underestimated by traditional measures of physiological stability.

A strength of this study was the use of statistical analysis to observe the interactional effects between the intervention and BGA or PMA. These interactional effects are important to consider because BGA is often associated with greater comorbidity (e.g., intraventricular hemorrhage, bronchopulmonary dysplasia) (Tan et al., 2021), and PMA is often associated with neurobehavioral maturity (McGowan et al., 2022). Because medical comorbidities and neurobehavioral maturity influence the infant’s physiological and pain response to sensory input, we would expect more stable and older infants to tolerate the ROP exam with fewer adverse events; however, there are a number of confounders that could influence the infant’s response including individual characteristics (White-Traut et al., 2004), infection (Walker, 2019), environmental noise and light (Hatfield et al., 2019), medications (Donato et al., 2019), and timing of exam as it relates to feeding and sleep (Tan et al., 2019).

Despite our own and previous reports (Disher et al., 2018; McCarty et al., 2019; Francisco et al., 2021) of variable pain responses to developmental interventions during eye exams, we recognize the well-defined role of physiological responses to pain (Boggini et al., 2021; Onuagu et al., 2022; García-Valdivieso et al., 2023). While our own study did not detect differences between group pain scores, we hypothesize that improvements in vital signs indicate potentially improved physiological responses to pain. Our study showed that the CEEM group appeared to reduce the number of desaturations to a greater degree for infants born at the earliest BGAs (e.g., 24 weeks) with attenuation of this effect with greater BGA. Regarding PMA, bradycardia and tachycardia events were reduced for infants across PMAs in the study, but the effect for tachycardia improves with age, while the effect for bradycardia diminishes with age. The CEEM group had reduced desaturation events, but only for infants at or near-term equivalent age (40 weeks PMA). While these trends are helpful for considering which infants are most likely to benefit from the CEEM, we must interpret with caution due to multiple possible confounders previously discussed [e.g., individual characteristics (Onuagu et al., 2022), environmental noise and light (Tan et al., 2021), medications (McGowan et al., 2022), and timing of exam as it relates to feeding and sleep (Brinkley et al., 2023)].

The majority of stakeholders had positive responses about the CEEM. Based on explanations provided in the survey, nurses especially perceived that infants had improved physiological responses and appreciated the improved efficiency of the CEEM afforded by the designated therapists that were part of the eye exam team; however, efficiency of the unit must be weighed against the additional staffing demands for the physical or occupational therapist’s allocation of time to eye exams as well as billing practices associated with skilled intervention.

## 5 Limitations

This study has several limitations. This is a nonrandomized, unblinded study of a small number of infants. Infants received an unequal number of examinations based on their medical need for ROP exams and the study period. Specifically, infants with lower BGAs have more visits than infants with older BGAs. While this unbalanced design was accounted for in our statistical analyses, multiple exams from some infants and only one for other infants may bias the data. Because the study was originally completed as a QI project, limited resources were available to conduct data collection; therefore, the number of outcome measures and length of the project were limited. Only one NICU intern was available to measure pain scores and exam time points (initiation of exam, exam duration, and exam end); therefore, time was documented based on the monitor display to align with vital signs during the period, which was less precise than use of a stopwatch. We enrolled all infants in the study regardless of respiratory support and did not differentiate between these infants.

Additionally, better delineation of particular developmental strategies used between groups is warranted for future investigations. Due to the quality improvement nature of this study, explicit notes about applications of developmental support in the SC group were not made. While developmental strategies employed during the 35 exams in the CEEM group were delivered consistently (as described in the methods section) by 2 trained and designated therapists, the developmental strategies during the 30 SC exams may have been delivered by as many as 30 separate nurses, all with different trainings in developmental care.

Due to the abovementioned limitations in data collection, we used mixed-effects models to account for the unequal number of eye examinations performed on the infants in the study. To aid in exploring the effects of the intervention on a population level, we opted to interpret the parameters of the non-random covariates and visualized their effects in the same fashion as we would have those from a marginal model (i.e., linear or generalized linear model). Due to the nature of the mixed-effects model, the interpretation of these parameters merits further scrutiny and caution. In short, these parameters would be interpreted in a marginal fashion only if the underlying patient population shared the same random intercept values. Therefore, mixed-effects models are typically used to model subject-specific outcomes (White-Traut et al., 2004). In reality, these individual random intercepts are not observable. Thus, the mixed-model would not adequately characterize the study outcomes on a population level. We caution the reader to take the findings on an exploratory basis, for this study serves as a foundation for more carefully planned follow-up studies, where balanced assignment to treatments, such as subjecting each infant to the same amounts of interventions, and more thorough data collection can allow us to model the outcomes using a marginal model.

## 6 Considerations for future studies

Due to variability in study methodology, outcomes used, and populations assessed (Disher et al., 2018), no consensus exists for nonpharmacological approaches to pain reduction during ROP exams in neonates. Future work should examine large, randomized cohorts of infants to allow stratification by age, respiratory support at time of exam, and other comorbidities. Furthermore, detailed descriptions of developmental interventions are necessary for reproducibility in future work and to reduce potential for bias.

While the majority of studies have used neonatal nursing staff to provide developmental support during the ROP exam (Rush et al., 2005), our study used trained neonatal physical therapists to provide developmental support. While both nurses and physical therapists working in the NICU have training specifically to address appropriate developmental care practices (Sweeney et al., 2010), studies have found that implementation of developmental care by nursing varies considerably based on professional efficacy (Mirlashari et al., 2019; Park and Kim, 2019), while developmental care is a core tenant of the neonatal therapist’s approach to practice (Khurana et al., 2020). While we did not appreciate a difference between nurse-provided developmental support (SC) and therapist-provided developmental support (CEEM) in many of the outcome variables measured, future work should consider the specific training of those providing developmental support on the quality of the interventions provided.

Additionally, future work should employ a more balanced design with equal visits per infant and should consider the use of outcome measures that might be more sensitive to infant responses including skin conductance, heart rate variability, vagal tone (Onuagu et al., 2022), and cortisol levels (Kleberg et al., 2008).

## 7 Conclusion

In conclusion, eye exams can be painful for vulnerable preterm infants. The CEEM, which incorporated best practices for preterm infant pain control, did appear to have influence on some vital sign parameters that varied based on infant BGA and PMA. Some significant interactional effects between the CEEM group and BGA and PMA, respectively were appreciated (number of desaturation events and probability of heart rate recovery lowered post-intervention for infants of same BGA; tachycardia and desaturation events increased post-intervention for infants of same PMA), indicating that infants of varying BGAs and PMAs may benefit differently from the intervention. The addition of neonatal therapists to the eye exam team was feasible and welcomed by stakeholders based on high rates of satisfaction with the CEEM for increasing efficiency of the eye exam process. Future research needed to determine clinical significance in larger cohort with more sensitive outcome measures and greater control of confounders impacting infant physiology and pain response.

## Data availability statement

The raw data supporting the conclusions of this article will be made available by the authors, without undue reservation.

## Ethics statement

The studies involving humans were approved by University of North Carolina at Chapel Hill IRB. The studies were conducted in accordance with the local legislation and institutional requirements. The Ethics Committee/Institutional Review Board waived the requirement of written informed consent for participation from the participants or the participants’ legal guardians/next of kin because initially conducted as a quality improvement project.

## Author contributions

DM: Writing – original draft, Validation, Supervision, Project administration, Methodology, Investigation, Conceptualization. EC-W: Writing – original draft, Project administration, Data curation. KL: Writing – original draft. TL: Writing – review & editing, Methodology, Formal analysis, Data curation. TZ-T: Writing – review & editing, Visualization, Data curation. NU: Writing – review & editing, Investigation. MG: Writing – review & editing, Supervision, Methodology, Investigation.
