# Supplementary material for: Interdisciplinary collaborative eye examinations to protect preterm infant neurodevelopment: a quality improvement project
Source: Front Psychol. 2024 May 6;15:1354033. doi: 10.3389/fpsyg.2024.1354033 (PMC11102993; doi:10.3389/fpsyg.2024.1354033)
Supplement: Supplementary file 1 [file Data_Sheet_1.docx]

Appendix A: Supplementary Table 1

Template for Intervention Description and Replication, (TIDieR) Criteria Applied to the Collaborative Eye Exam Model (CEEM) Intervention

| BRIEF NAME |  |
| --- | --- |
| Provide the name or a phrase that describes the intervention. | Collaborative Eye Exam Model - CEEM |
| WHY |  |
| Describe any rationale, theory, or goal of the elements essential to the intervention. | CEEM is a coordinated, multidisciplinary effort to reduce infant pain and optimize physiologic state during routine retinopathy of prematurity (ROP) screening exams. CEEM involves neonatal physical and occupational therapists providing behavioral and physiologic state supports before, during, and after the infant’s ROP exam with an ophthalmologist. |
| WHAT |  |
| Materials: Describe any physical or informational materials used in the intervention, including those provided to participants or used in intervention delivery or in training of intervention providers. Provide information on where the materials can be accessed (e.g. online appendix, URL). | The therapists used positioning aids at bedside or immediately available to contain infant. These items include blanket swaddles, rolled blankets and weighted bean bags, and conformable pillows. Therapists also used pacifiers and sucralose. |
| Procedures: Describe each of the procedures, activities, and/or processes used in the intervention, including any enabling or support activities. | Prior to exam:   - Two neonatal therapists coordinate with ophthalmology to determine a logical schedule for infants to receive ROP exams based on ophthalmologist, therapist, and infant availability as well as infant location within unit. - Neonatal therapists (NT) complete chart review and coordinate with bedside RN for assigned infants to determine any additional medical supports that may be appropriate (RN may stop feedings, increase oxygen support, etc.). - All infants scheduled for ROP screening receive dilating drops 1-2 hours prior to examination, typically administered by bedside RN.   Set up:   - NT arrives to infant’s bedside a few minutes prior to infant’s exam to prepare and position infant. NT provides swaddling, age appropriate state supports (e.g., swaddling, dimming lights), and gradual transition to optimal position for exam to reduce infant stress associated with repositioning. - Ophthalmologist and NICU intern arrive to infant’s bedside. Ophthalmologist administers topical anaesthetic.   During exam:   - NT provides containment via swaddle, positioning aids (if appropriate and at bedside per PMA) and via “hand hug” across infant’s upper body to reduce motor reactivity and associated stress cues. NT provides pacifier and oral sucralose if medically appropriate as well as calming auditory input to reduce infant experience of pain. NT provides containment via head cup and adjusts cervical position as directed by ophthalmologist to expedite exam. NT monitors infant’s vital signs and suggests providing a break if infant experiences bradycardia, tachycardia, or desaturation that does not recover. - Ophthalmologist provides standard ROP screening. - Bedside RN remains available in room to intervene if necessary.   After exam:   - NT remains at bedside with infant to provide calming strategies and repositioning to promote return of behavioural and physiologic state to baseline. - NT discusses exam and infant tolerance and positioning with bedside RN. - Bedside RN makes any necessary adjustments to infant’s oxygen, feeding, or other medical supports. - Ophthalmologist departs bedside and continues to the next infant scheduled, where the 2^nd^ NT has already prepared and positioned the next infant. |
| WHO PROVIDED |  |
| For each category of intervention provider (e.g. psychologist), describe their expertise, background and any specific training given. | Neonatal Therapist:   - Neonatal therapists with a doctoral degree in physical therapy. - Two qualified pediatric ophthalmologists.   Medical Providers:   - RN and Respiratory Therapists immediately available in unit. |
| HOW |  |
| Describe the modes of delivery (e.g. face-to-face or by some other mechanism, such as internet or telephone) of the intervention and whether it was provided individually or in a group. | CEEM is a face-to-face, hands-on intervention provided to individual infants participating in routine ROP screening. |
| WHERE |  |
| Describe the type(s) of location(s) where the intervention occurred, including any necessary infrastructure or relevant features. | Intervention occurred at the infant’s bedside in a Level IV NICU. |
| WHEN and HOW MUCH |  |
| Describe the number of times the intervention was delivered and over what period of time including the number of sessions, their schedule, and their duration, intensity or dose. | Intervention was provided during routine ROP screenings. ROP screenings were typically scheduled one day per week for a period of 1-2 hours starting between 7:30am and 10:00 depending on caseload and ophthalmologist availability. Individual infants were typically scheduled weekly or bi-weekly for exams based on clinical factors including age and any prior ROP exam findings. |
| TAILORING |  |
| If the intervention was planned to be personalised, titrated or adapted, then describe what, why, when, and how. | Intervention was tailored to individual infant response including stress cues and changes in vital signs. NTs adapted containment and calming strategies to each infant.  Intervention, including positioning before and during the exam, was also adapted based on the needs of the ophthalmologist to improve the efficiency of exam so as not to prolong infant exposure to noxious stimuli. |
| MODIFICATIONS |  |
| If the intervention was modified during the course of the study, describe the changes (what, why, when, and how). | No modifications made to the structure, sequence, or content of the CEEM intervention. |
| HOW WELL |  |
| Planned: If intervention adherence or fidelity was assessed, describe how and by whom, and if any strategies were used to maintain or improve fidelity, describe them. | Two designated therapists employed the developmental intervention. The PI (DM) developed the intervention and trained the second interventionist (EC-W) in the CEEM for period of 6 weeks prior to study initiation. |
| Actual: If intervention adherence/fidelity was assessed, describe the extent to it was delivered as planned. | N/A |

**Appendix B.** Stakeholder Survey for Collaborative Eye Exam Model

This survey is regarding the addition of neonatal therapy to retinopathy of prematurity (ROP) eye examinations in UNC’s Newborn Critical Care Center. Please describe your experience with the addition of therapist assistance during examinations. We would love your feedback!

How did you feel the addition of neonatal therapy was for the infant’s experience?
5 – very beneficial
4 – somewhat beneficial
3 – neither beneficial nor detrimental
2 – somewhat detrimental
1 – very detrimental

How did you feel the addition of neonatal therapy changed the infant’s experience?
4 – very much improved
3 – somewhat improved
2 – no change
1 – detrimental
0 – N/A, not observed

How did you feel the addition of neonatal therapy changed the staff’s experience?
4 – very much improved
3 – somewhat improved
2 – no change
1 – detrimental
0 – N/A, not observed

How did you feel the addition of neonatal therapy changed the parent’s experience?
4 – very much improved
3 – somewhat improved
2 – no change
1 – detrimental
0 – N/A, not observed

What benefit(s) did you experience with the addition of neonatal therapy to ROP exams? Please describe any here.
__________________________________________________________________________________________________________________________________________________________________________________________________________________________________________________________________________________________________________________

# Did you experience any drawbacks or difficulties with the addition of neonatal therapy to ROP exams? Please let us know what can be improved! _________________________________________________________________________________________________________________________________________________________________________________________________________________________________________________________________________________________
